# Post-Graduate Study

**Published:** 1887-11

**Authors:** 


### Editorial.

### POST-GRADUATE STUDY.

Atlanta is to have a Post-graduate Medical School.

During the past four or five years there have been numerous schools established in this country for the purpose of giving post-graduate instruction to medical men.

Schools of this kind have long been in operation in the cities of Europe, and it is remarkable that the physicians of America have only recently felt the need of them. That such a need has finally been felt and appreciated is attested by the numerous post-graduate schools which have sprung up in a few years and by the wonderful success which they have attained.

New York took the lead in this matter, and her example was speedily followed by nearly all the large cities.

The aim of all these schools is the same, viz., to give practical and clinical instruction on such subjects as are not thoroughly taught in a college curriculum. The need of this kind of teaching is felt by two classes of physicians. In the first place, the young graduate, whose breast still swells with pleasure as he looks on his new diploma, is keenly aware of the faɔt that most of his knowledge has been drawn from text-books, and he would fain have a more intimate acquaintance with disease before assuming the heavy responsibilities of a practicing physician. In the second place, the physician who has been in practice for a number of years finds that he is gradually dropping behind the times, and is glad to avail himself of a few months' practical study in a large city this enables him to learn all that is new in the various branches of medicine and surgery. These two classes of men crowd the post-graduate schools, eager to learn all they can.

Atlanta is undeniably the medical centre of the South, and it justly deserves this distinction. Its advantages briefly stated are these: It is the largest city in the South, with the exception of New Orleans ; it is the greatest railroad centre in the South, making it easy of access from all points ; it is healthy all the year around, and being on an elevated plateau over 1,000 feet above the sea, it is free from epidemic diseases ; it has for thirty years been noted for its distinguished medical teachers ; it has an abundance of clinical material ; and lastly, it is a cheap place to live in.

These advantages render Atlanta a peculiarly fit place for a post-graduate school, and it is with pleasure that we chronicle the fact that the organization of such an institution is about complete. All the chairs have not yet been filled, but we are assured that none but competent teachers in the various branches of medicine will be selected. It is highly probable that the faculty of the new school will secure rooms for lecture and clinical purposes in the Atlanta Medical College.

THE JOURNAL extends its best wishes to the post graduate school, and predicts for it a successful and useful career.

